# Influence of Long-Distance Climate Teleconnection on Seasonality of Water Temperature in the World's Largest Lake - Lake Baikal, Siberia

**DOI:** 10.1371/journal.pone.0014688

**Published:** 2011-02-16

**Authors:** Stephen L. Katz, Stephanie E. Hampton, Lyubov R. Izmest'eva, Marianne V. Moore

**Affiliations:** 1 Channel Islands National Marine Sanctuary, National Oceanographic and Atmospheric Administration, Santa Barbara, California, United States of America; 2 National Center for Ecological Analysis and Synthesis, University of California Santa Barbara, Santa Barbara, California, United States of America; 3 Scientific Research Institute of Biology, Irkutsk State University, Irkutsk, Russia; 4 Department of Biological Sciences, Wellesley College, Wellesley, Massachusetts, United States of America; University of California San Diego, United States of America

## Abstract

Large-scale climate change is superimposed on interacting patterns of climate variability that fluctuate on numerous temporal and spatial scales—elements of which, such as seasonal timing, may have important impacts on local and regional ecosystem forcing. Lake Baikal in Siberia is not only the world's largest and most biologically diverse lake, but it has exceptionally strong seasonal structure in ecosystem dynamics that may be dramatically affected by fluctuations in seasonal timing. We applied time-frequency analysis to a near-continuous, 58-year record of water temperature from Lake Baikal to examine how seasonality in the lake has fluctuated over the past half century and to infer underlying mechanisms. On decadal scales, the timing of seasonal onset strongly corresponds with deviation in the zonal wind intensity as described by length of day (LOD); on shorter scales, these temperature patterns shift in concert with the El Nino-Southern Oscillation (ENSO). Importantly, the connection between ENSO and Lake Baikal is gated by the cool and warm periods of the Pacific Decadal Oscillation (PDO). Large-scale climatic phenomena affecting Siberia are apparent in Lake Baikal surface water temperature data, dynamics resulting from jet stream and storm track variability in central Asia and across the Northern Hemisphere.

## Introduction

Shifts in both magnitude and timing of temperature, precipitation and other climate variables associated with climate change have affected ecosystems, independently and in concert. Alterations in productivity and species ranges have been correlated with rising temperatures, and phenological changes have been evident as the timing of seasonal events has shifted across ecosystems [1]–[3]. It is increasingly appreciated that shifting abiotic and biotic seasonality manifests across a broad range of temporal scales related to climate variability, in addition to those associated with long-term warming, with cascading repercussions for ecosystems [1], [4]–[6]. Applying the tools of signal processing to evaluate patterns of seasonal variability in climate and local ecology may reveal important messages about how these systems are connected across space and what ecosystem level consequences may be expected.

Seasonal timing is defined in various ways across ecosystems and individual studies. Many ecologists define seasonal transitions by identifying biological threshold temperatures to be crossed [7], [8] and in the case of aquatic systems, the onset and deterioration of thermal stratification [9], [10] can be a useful seasonal indicator. Stine et al. [11], however, emphasize that the use of such thresholds in defining season can conflate changes in timing of season with changes in the annual mean, and these authors exploited an implementation of spectral analysis to describe season in time series data. In spectral analysis, the temporal positions of the harmonics (including harmonics with an annual frequency) contributing to the observed dynamics of a time series – phase (*Φ*) – are estimated over the length of the time series. If seasonal signals are non-stationary, in that they vary across a time series [12], the locally estimated phases will deviate from the phase estimated for the entire time series and the deviation can be expressed as a relative phase. For example, by examining patterns in the relative phase discerned from Fourier transform of sequential, short environmental time series, long term trends in air temperature seasonality from diverse locations have been documented in the climate literature [13], and Stine et al. [11] demonstrated that earlier seasonality in Northern Hemispheric air temperatures is related to changing atmospheric gas composition.

These previous studies of variability in seasonality focused on decadal to centenary-scale drivers of the seasonal variation that is described by phase in spectral analysis, but this analytical approach also offers the opportunity to examine shorter-scale variability that is frequently more relevant to ecologists and others examining local environmental data. To detect connections among time series, such as local temperature and climatic drivers, the covariance of informative anomalies must be discerned with high temporal resolution (e.g. monthly), and with sufficient duration (e.g. multi-decadal) such that a diversity of behavior is observed. Few environmental monitoring programs provide data that satisfy these criteria.

Since 1946, a limnological monitoring program on Lake Baikal in Siberia has produced a high quality data set [14] that meets these criteria and provides a unique record of large-scale environmental change in one of the most rapidly changing regions on the planet [15]. Physical (water temperature and clarity) and biological (plankton abundance and species composition) data have been collected on approximately biweekly intervals and through all seasons of the year from a single location on the lake 2.7 km offshore from the southwest coast [16]. Holding 20% of the world's liquid fresh water, this massive water body can be expected to exhibit strong thermal inertia such that temperature changes in the lake clearly indicate large-scale regional phenomena [16]. Importantly, Lake Baikal is also uniquely positioned to integrate effects of jet stream and storm track dynamics traversing Asia, as it lies at the nexus of the influential Siberian High. Seasonal abiotic changes are particularly interesting in Lake Baikal because seasonal contrasts in biotic communities are so strong here [16], [17] with cold-adapted endemic biota giving way to cosmopolitan taxa during warm periods.

Using these data from Lake Baikal, Hampton et al. [16] showed that annual surface water temperatures increased 1.2°C since 1946, and the variance of these water temperature data was non-stationary (i.e., the variance of a time series constructed of quarterly-averaged values was not independent of position in time). In that study, deviations from stationarity were treated as a nuisance, requiring a special approach to data filtering. To remove the annual seasonality, the time series was transformed to the frequency domain in short segments using Short-Time Fourier Transforms (STFT; [18]), where the annual harmonic was reduced to background noise levels, and then inverse transformed to the time domain.

**Figure 1:**
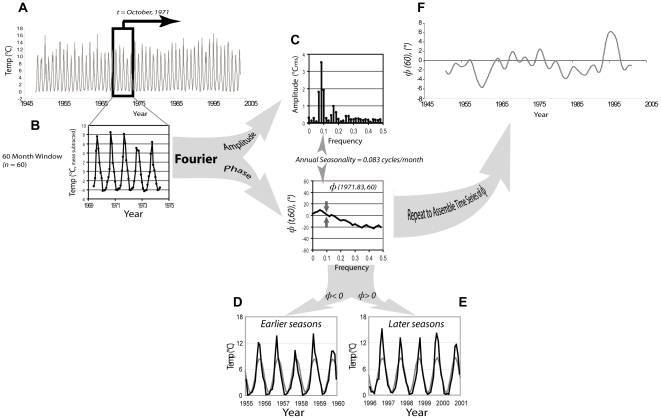
Workflow for estimating phase and creating a phase time series. (A) Extraction of annual seasonality anomaly (*Φ*(*t*,n)) from long term Lake Baikal surface temperature time series. (B) The time series is mean-subtracted and then a sliding window of varying length, in this case 60 months (*n* = 60), is extracted sequentially as the window is passed down the length of the series. (C) At each point in time (*t*), the small window is Fourier transformed and the magnitude and phase spectra of the harmonic components are estimated. The annual seasonality is the peak at 0.083^ ^^ cycles month^−1^, and *Φ*(1971.83^ ^^,60) is, in this example, the phase of the annual harmonic for a window 60 months long, centered at October 1971. (D and E) Time series of lake temperature (gray) compared to a single annual harmonic with a single phase estimated for the entire 58 year time series (black) to indicate the local phase anomaly, with examples of “early” and “late” seasons indicated by phase. The 60 month period 1955–1960 (D) has negative *Φ* values, indicating that lake temperature variations are advanced relative to the long term seasonality. This is observable as particularly cold Fall seasons (water temperature falling). The 60 month period 1996–2001 (E) has positive *Φ* values, indicating that lake temperature variations are delayed relative to the long term seasonality. This is observable as particularly cold Spring seasons (water temperature rising). (F) Time series of *Φ*(*t*,60) assembled by repeatedly estimating the phase of the annual harmonic as the 60 month window is passed down the data set. This time series would then be pre-whitened (noise addition) to reduce auto-correlation for cross-correlation analysis.

The deviation from regular seasonality, however, is an anomaly that we explicitly examine here as a potential indicator of important connections between local conditions and large-scale climate teleconnections. Specifically, we focus on the information relayed by the relative phase of the annual harmonic [11], extracted from the magnitude and phase spectrum of the time series of transformed monthly mean temperatures (*Φ*; Fig. 1), which can be interpreted as the timing of the onset of seasons in the lake. Mechanistically, autumnally decreasing lake surface water temperature is the consequence of the combined influence of an increasing thermal gradient between the air and water and increased seasonal northwest winds that disrupt lake stratification [17], [19]. Stratification in Lake Baikal persists weakly for about 4–6 weeks of the summer and is readily disrupted by strong winds and storms [19].

To understand not only how seasonality in Lake Baikal has fluctuated over the past half century, but also to infer underlying mechanisms, we examined the relationship of phase with a suite of climate drivers that together describe the trajectory and force of the jet stream delivering weather systems to Lake Baikal. Length of Day (LOD) is a proxy for changes in the angular velocity of atmospheric zonal winds [20], an index that should yield information about the total energy that may be available for storm systems as the jet stream reaches Siberia. The Arctic Oscillation (AO) describes pressure systems across the Atlantic Ocean that may affect jet stream dynamics as zonal winds approach Eurasia from the West [21]. The Pacific Decadal Oscillation (PDO) and El Niño Southern Oscillation (ENSO) provide information about pressure systems that influence the jet stream trajectory as it exits the continent at the Pacific Ocean. Ultimately we are able to suggest a pathway through which global climatic activity affects major seasonal transitions in Asia and Lake Baikal.

## Results and Discussion

**Figure 2:**
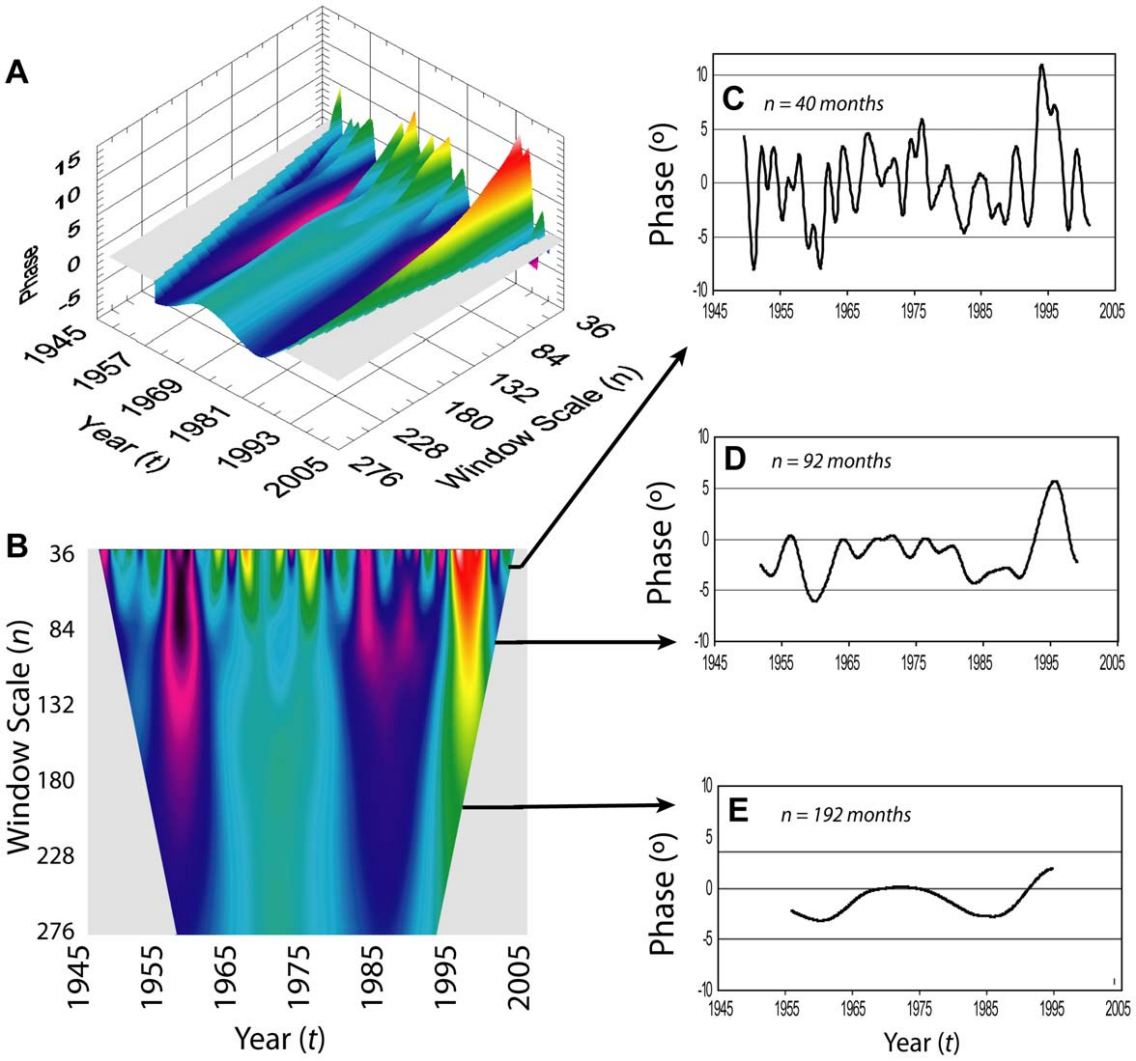
The values of *Φ*(*t*,*n*) over a range of frequency scales and across the time series from 1948 to 2004. (A and B) Assembled values of *Φ*(*t*,*n*) from long term Lake Baikal surface temperature time series over the period 1948 to 2003 and window sizes (*n*) 36 to 276 months. Cooler colors are more negative *Φ*(*t*,n); warmer colors are more positive *Φ*(*t*,*n*); if phase were a constant, the surface would be flat. The surface tapers with increasing *n* as *Φ*(*t*,*n*) is referenced to the center of the window of length *n*, and as *n* increases there is an increasing *n*/2-long segment that does not have a relevant measure of *Φ*(*t*,*n*) at the beginning and end of the time series. (C, D and E) Time series of *Φ*(*t*,*n*) sampled at *n* equal 40 (C), 92 (D) and 192 (E) months. Estimating *Φ*(*t*,*n*) at longer *n* averages over a longer time and is equivalent to smoothing the low *n Φ*(*t*,*n*) time series. As such *Φ*(*t*,*n*) at lower values of *n* captures temperature anomalies occurring at higher frequencies and vice versa.

Variation in seasonality of lake water temperatures, estimated over varying windows of time (*n*) across the time series (*t*) (Fig. 1) reveal several important temporal patterns that are illustrated in a surface plot of relative phase *Φ*(*t,n*) (Fig. 2 A&B). In this case, the range of *n* is displayed from 36 to 276 months, as the STFT window slides from *t* = 1948 to 2002. The first observation is that the time series is not stationary and that *Φ*(*t,n*) in fact varies. Warmer colors indicate higher phase values corresponding to delayed seasonality; cool colors express negative phases corresponding to advanced seasonality. It is important to note that the total range of values is 21 degrees of phase — from −8.8° in late 1950 to 12.2° in 1994 (Fig. 2C). Thus, the extent of variability in annual seasonality is small (6% ∼21.3 days/year) – generally less than the 2-week average sampling interval. Superficially the variability at small *n* shows little resemblance to the variability at large *n*, which estimates phase over a longer interval. However, by plotting the data as a continuous surface it is clear that the large scale features in the time series – negative phase in 1960 and 1985 (i.e. earlier seasons), and positive phases in 1970 and 1995 (i.e. later seasons)—are consistent across scales.

The Lake Baikal data assembled in this way can be sampled to evaluate dynamics occurring at both short and longer time scales. The longest time scale over which water temperature varied was the linear, long-term warming trend over the entire 58 year time series reported previously [16]. Previous spectral analysis of Lake Baikal surface temperatures revealed notable periodicities at 2–6, 14–16, and 28–36 years [19]. These published observations provided a starting point to examine shorter time scales (i.e., *Φ*[*t*,40 months], Fig. 2C; *Φ*[*t*,192 months], Fig. 2E ) within these data for relationships with climatic indices known to influence weather systems at scales likely to be relevant for phenological change [1], [5], [22], [23].

**Figure 3:**
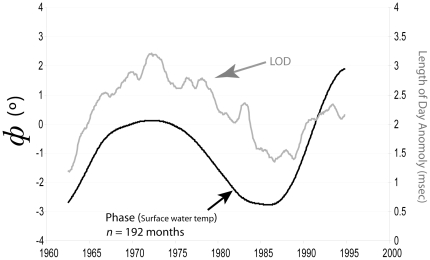
Annual seasonality phase anomaly (*Φ*(*t*,192)) plotted with the deviation in length of day (LOD). LOD is presented as smoothed values where the variances contributed by harmonics higher than an annual period were lowered to match the raw *Φ*(*t*,192) time series (see Methods). The strong relationship suggested by visual inspection is supported by a high maximum cross-correlation coefficient between pre-whitened time series of 0.507 with no lag. Data were continuous from January 18^th^, 1963 to December 31^st^, 2003 (40 years).

LOD is a reflection of planetary angular momentum and variability in LOD expresses changes in the angular velocity of atmospheric zonal winds [20]. As the atmosphere heats and accelerates zonal winds, the solid planet slows down to maintain the constant total angular momentum of the solid, fluid and gas components of the Earth. Zonal wind intensity varies on several temporal scales, with long term changes consistent with models of global heating [24], [25], and changes at shorter time scales that may indicate climate regime shifts [26]. We discovered a significant relationship of the LOD anomaly with phase at the broadest scale, *Φ*(*t*,192), with a cross-correlation coefficient of 0.507 at a lag of zero months (*p*<0.0001; Fig. 3), for the period where both data sets are available (1962–1994). It is apparent on inspection of Fig. 2 that the features of *Φ*(*t*,192) that correlate with LOD are conserved at a range of window sizes (*n*), although this correlation modestly declines in magnitude as *n* deviates from 192 months. For example, in a window of 92 months the coefficient ranged from 0.38 to 0.41 for lags plus and minus 12 months. In addition, when we removed the long-period seasonality apparent in the LOD time series (ca. 25-yr), and correlated the residuals with *Φ*(*t,n*) at small values of *n*, no statistically significant relationship could be discerned. Thus, the surface water temperature in Lake Baikal transduces the LOD anomaly, but principally on the longer 16-yr (*n* = 192) time scale of variability.

**Figure 4:**
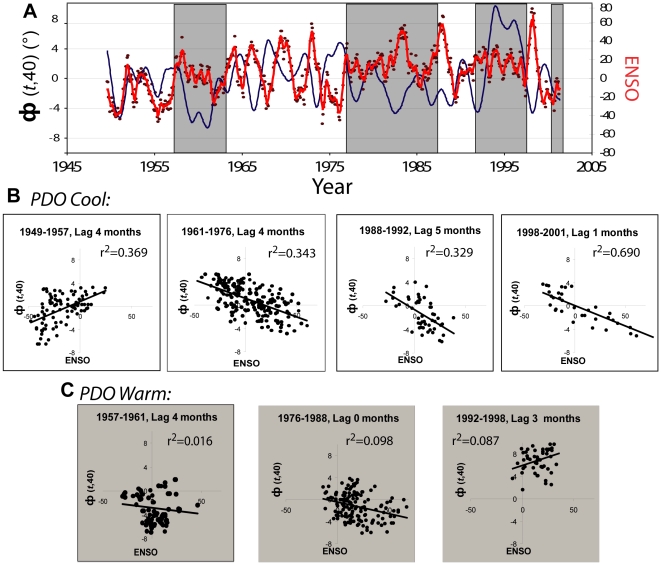
The temporal relationships and correlation structures of *Φ*(*t*,*n*) and the Global-SST ENSO index across the history of the PDO index from 1946 since 1946. (A) Plot of monthly *Φ*(*t*,40) time series (Blue) with the monthly Global-SST ENSO index (Red) from 1946–2004. The Global-SST ENSO index captures the low-frequency components of other, location specific ENSO indices. The data were continuous from January 1946 to December 2003. Global-SST ENSO is presented as raw, unfiltered values (points) and smoothed to produce similar high frequency contributions to *Φ*(*t*,40) (line) (see Methods). The periods 1957–1964, 1976–1988 and 1992–1998 are PDO “warm” phases and are indicated in gray, the intervals are PDO “cool” periods. (B and C) Plots of the Global-SST ENSO index plotted against *Φ*(*t*,40). Data were pre-whitened to reduce autocorrelation for analysis. Cross-correlation coefficients of determination (displayed) were estimated for the entire periods indicated. Data are plotted at the lag that produced the greatest cross-correlation coefficient magnitude to convey the strength of the relationship. The mean cross-correlation coefficient (*r*) for the PDO cool periods (B) was 0.67 at an average lag of 3. 5 months with ENSO leading *Φ*(*t*,40).

At shorter time scales (smaller *n*), other climate teleconnection patterns were evident in the Lake Baikal temperature data, but with complexity involving interplay between ENSO and the PDO indices. Figure 4A is a plot of the raw *Φ*(*t*,40) and Global-SST ENSO anomaly time series. The raw monthly data have a significant level of first-order autocorrelation, and were pre-whitened (see Methods) to prevent autocorrelation from biasing the cross-correlation estimates. The largest cross-correlation coefficient (*r*) between pre-whitened *Φ*(*t*,40) and this ENSO index over the entire period of 1946–2002 was −0.10 when the ENSO index leads the *Φ*(*t*,40) by 4 months. This relationship was not statistically significant (*t_s_* = 2.44; *p* = 0.015) in the context of a Bonferroni adjustment of type-I error rate (α_a_ = 0.002; see methods). Further inspection revealed that the nature of this relationship changed across the time series, with an apparent, stronger relationship in the 13 years preceding 1976. The year 1976 is now recognized as a transition point in the PDO [27]. The spatial footprint of PDO is similar to Global-SST ENSO, but with a very different pattern of temporal variability, alternating between “warm” and “cool” phases on decadal scales. Specifically, the PDO was in a warm phase during the periods from 1957–1961, 1976–1988 and 1992–1998 with the intervening periods defined as cool [28], and some have suggested that 2002 brought another transition from cool to warm [29]. Using the PDO's warm and cool phase transitions to define segments of the ENSO and *Φ*(*t*,40) time series *a priori*, we found very strong cross-correlations (*r*) during PDO cool phases, with no cross-correlation coefficient magnitude smaller than 0.57 (all *p*<0.0001), with the strongest relationships when ENSO is an average of 3.5 months ahead of *Φ*(*t*,40) (Fig. 4B). During the PDO warm phases, no consistent correlations were observed, with no correlation coefficient magnitude larger than 0.27 at any lag (Fig. 4C). Thus, the Lake Baikal surface temperature time series was shown to transduce ENSO – an index of sea surface temperature anomalies 10,000 km away–but only when the PDO was in its cool phase, and this teleconnection was deciphered in the short-term (*n* = 40) variability in annual seasonality. Other studies have shown interactions between ENSO and PDO where climate forecasting predictability during La Niña or El Niño episodes was affected by PDO status, with periods of greater or lesser predictability in both PDO conditions [30], [31]. It seems likely that increasing the resolution of observational studies may motivate a more structured physical model of local climate and further improve local forecasts for the central Siberian region.

While pre-whitening is useful as a technique to prevent autocorrelation from biasing the estimates of cross-correlation, it is important to remember that the autocorrelation in the original time series is additional information, not just nuisance, and there are circumstances where we would like to access all available information. Forecasting is a situation in which the temporal autocorrelation in data is useful information that should be retained. As described above, we have observed statistically significant relationships between *Φ*(*t*,40) and an ENSO index with autocorrelation removed, and so inferred that a physical relationship is likely to exist. Retaining the autocorrelation in the original data now allows us to evaluate how much of the variability in phase *Φ*(*t*,40) could be explained by the ENSO index data, and therefore useful for forecasting the onset of seasons. During cool PDO periods, coefficients of determination (*r^2^*) of cross-correlations between raw *Φ*(*t*,40) and ENSO indices ranged from 0.45 and 0.59 with ENSO leading *Φ*(*t*,40) by an average lag of 3.75 months, compared to 0.19 to 0.45 for the pre-whitened (autocorrelation removed) data. During warm periods, the *r^2^* for the same cross-correlation ranged from 0.01 to 0.07 with ENSO leading *Φ*(*t*,40) by approximately three weeks on average. Thus, during PDO cool periods, 45% to 59% of lake temperature seasonality variability can be accounted for by the variability in ENSO three to four months in advance. The ability to anticipate early and late winters with such strong confidence is particularly important in a region where environmental conditions are so severe. It is exciting to contemplate the potential improvements in season-scale forecasting that are possible when the dynamics of the system are more fully explored.

Inspection of Figure 4 presents two additional questions. First, what does the sign change in the ENSO-*Φ*(*t*,40) relationship before and after 1957 tell us about the process underlying the observed relationships? Second, is Lake Baikal *Φ*(*t*,40) “listening” to some other climate variability signal during the PDO warm phases?

**Figure 5:**
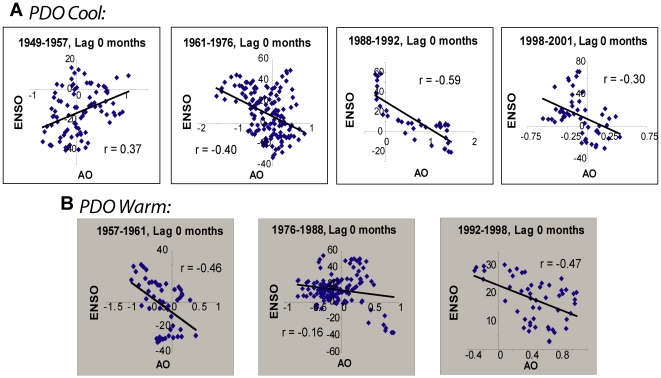
Plots of the Arctic Oscillation (AO) against Global-SST ENSO index across the history of the PDO index since 1946. Data were pre-whitened to reduce autocorrelation. Cross-correlation coefficients (displayed) were estimated for the entire periods indicated. Data are plotted at a lag of zero months, which maximized the cross-correlation coefficient magnitude in order to convey the strength of the relationship.

A partial answer to the first question is suggested by evaluating the relationship between monthly ENSO and AO indexes. AO is known to influence Asian sub-polar jet stream flow and storm-tracks [32], [33] and specifically the intensity of the Siberian high [21], and is thus a candidate for communicating information to the Baikal region. This relationship is not simple however, and AO variability may be more strongly associated with temperatures than with surface winds as is the case in East Asian monsoons [34]. Though statistically significant, cross-correlations between ENSO and AO index time series were not particularly impressive. Cross-correlation coefficient magnitudes ranged from 0.16 to 0.59 (0.04>*p*>10^−5^), with maximum values coinciding with zero lag between the time series (Fig. 5). However, in the period 1946–1957 the relationship between ENSO and AO was positive, and thereafter the relationship was negative if it existed at all. It has been noted previously that 1957 was a moment of significant change in phase between the North Atlantic Oscillation (NAO) and North Pacific Index (NPI) [35], indices that correlate with the AO and ENSO respectively. Thus, it is not a complete surprise that the sign of the AO-ENSO relationship might also change in 1957.

**Figure 6:**
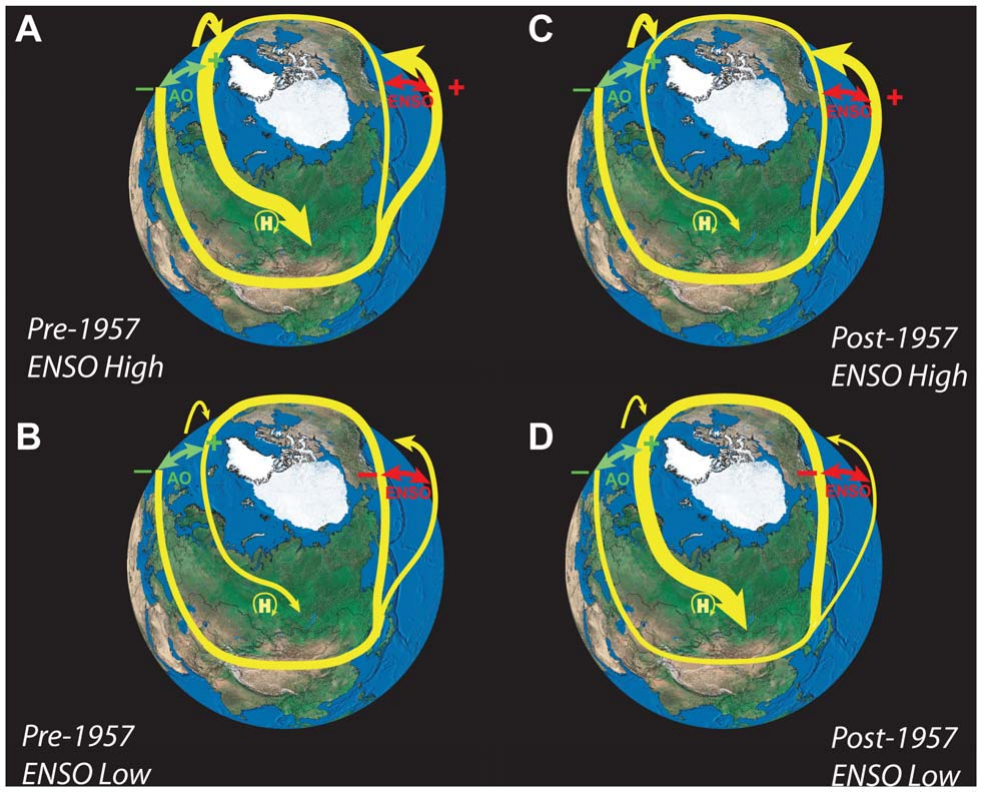
Suggested interaction between ENSO and AO affecting jet stream (JS) trajectories (yellow) over central Asia in the periods before 1957 and from 1957–2003 (C&D) during PDO “cool” phases. Prior to 1957 positive variations in ENSO (red arrow), correlated with stronger, persistent lower latitude sub-tropical JS flow and less energy driven up into the polar jet across the Pacific (A&B). These conditions were positively correlated with *Φ*(*t*,40) because of strong, northerly polar JS into central Asia consequent to positive AO values (green arrows). In periods of low ENSO values, the converse would prevail, with JS flow diverted higher latitudes on exit from the Asian Far East. In the period following 1957, AO and ENSO are out of phase and negative values of ENSO are associated with more energy pushed to higher latitudes across the Pacific and more variable, stronger polar jets arriving at the Atlantic. These periods are correlated with lower AO indexes and minimal additional energy pushed to higher latitudes and stronger flow arriving in central Asia, with the converse in periods of low ENSO index. In either case, (high-AO pre-1957, low-ENSO post-1957) strong polar JS flow arriving at the Siberian high (H) is diverted in an anti-cyclonic direction across Lake Baikal producing more negative *Φ*(t,40), colder fall water temperatures and earlier winter conditions.

While we do not know the mechanistic basis of this change in relationship between AO and ENSO in 1957, the change itself in the context of other published findings generates a hypothesis regarding the manner in which large scale patterns of climate variability communicate across tens of thousands of kilometers, ultimately to be evidenced in the Lake Baikal data (Figure 6). Positive AO anomalies predict a strong northward polar jet stream as it crosses Scandinavia and enters central Asia, while negative AO anomalies predict strong southern trajectories for the sub-tropical jet stream over the Iberian peninsula and entering central Asia [22]. On the eastern side of the continent, ENSO predicts the jet stream trajectory as it leaves central Asia. Low ENSO anomalies are associated with northward locations of the North Pacific High and blocking of the sub-tropical jet stream as it exits central Asia, while high ENSO anomalies are associated with the jet exiting central Asia at low latitudes [36]. Low (high) ENSO indices have also been correlated with stronger (weaker) Siberian high pressure center, stronger (weaker) jet stream flow, more (less) frequent cold surges spawned southeasterly across East Asia including Lake Baikal, and more (less) intense surface wind events [37]. Even so, Zhang at el. [37] pointed out that the relationship between jet stream strength and the intensity of the Siberian high is neither monotonic nor simple. During particularly strong jet stream periods, short wave events that might otherwise spawn cold surges into the Baikal region early in the winter (i.e. December) pass through the large scale system without doing so [37], [38]. This proposed scenario is supported by recent work demonstrating significant correlations between ENSO and cold surges arriving in the Asian far east [39]. Strong surface winds that accompany cold surges can disrupt lake stratification [17], and a reduction in these surface winds in December could produce a positive *Φ*(*t*,40), or delayed seasonality in surface water temperature. On the other hand, less extreme jet stream intensities do spawn cold surges in the Baikal region early in the winter [37] resulting in a more negative *Φ*(*t*,40), but seemingly only during PDO cool periods (Fig. 4).

This interannual variability in jet stream trajectory expressing the impacts of climate teleconnection is superimposed on a mean jet stream flow that can be described as a converging spiral centered on the North Pole [40]. On this average spiral pathway, the subtropical jet leaves east Asia continuing along a meandering path, with kinetic energy diverted to higher latitudes until the polar jet arrives in central Asia to be diverted southward, along with winter storm tracks, by the anticyclonic Siberian high [37], [41]. This scenario is depicted in Figure 6; as the jet stream path meanders and bifurcates under the influence of meridional pressure gradients (expressed in ENSO and AO), the energy in the polar jet will vary as it arrives in Siberia.

The observed suite of relationships suggests that *Φ*(*t*,40) in the Lake Baikal data responds to the energy in the polar jet stream. The variability of the jet stream's local and regional influence is, in turn, directed by the balance of pressure and temperature dynamics in the North Atlantic and Pacific Oceans. Importantly, examination of longer and shorter windows in the STFT showed a rapid degradation of the relationship with ENSO, and no similar relationship was observed with AO or other teleconnection index at other values of *n*. Given this proposed dynamic, with kinetic energy transmitted over such great distances, it is reasonable that the cross-correlation coefficients suggested a several month lag between events in the Pacific and at Lake Baikal.

Jet stream variability has been recognized as a powerful forcing agent on local water dynamics such as oceanic upwelling [42], and the present findings suggest similar relationships in lake Baikal. However, even with statistically significant correlation coefficients, there is a lot of unexplained variance attributable to other forcing and the influence of local conditions. Shimaraev and colleagues [19] suggested that lake temperature dynamics were critically dependent on local atmospheric-water coupling. In particular, those authors argued convincingly that temperature-driven local winds were critical in driving heat flux across the lake surface. Indeed, while it would have been satisfying to express our findings in terms of the local winds, the observational data are sparse and where wind data exist, they are at wide variance with reanalysis modeling of surface winds [43]. Lake Baikal is bounded on the west by ridges of the Baikal and Primorskiy mountains that are 2000–3000 m high 2–4 km from the lake's shore. This geography results in prevailing westerly winds traveling down to the lake via eddies and counter flow that result in wind dynamics not well predicted by re-analysis models. In addition, the sub-tropical jet stream itself is not so much a coherent jet as an average flow with many scales of turbulence [44]. Dynamic interactions of strength, and small- versus large-scale wave propagation, have been described for this system [38], [37], [45]. If the structural characteristics of the jet stream turbulence also vary between PDO phases, due to net forcing in these periods [46], it is possible that energy exchange from jets near the tropopause down to the surface at Lake Baikal will likewise vary between PDO phases. Such a relationship between the jet stream strength or scale of turbulent structures and the PDO would help explain the observed gating between ENSO and Lake Baikal water temperatures. In any event, while Lake Baikal may be at the intersection of large-scale climate teleconnections on various scales at different times, these large scale drivers will always be filtered through local and regional dynamics to produce the observed lake conditions.

Examination of the long-term temperature time series has revealed a spectrum of connections between planetary-scale climate features and surface lake temperatures monitored over 58 yrs. This surface temperature time series has revealed on its longest time scale a rapid warming in the surface waters of Lake Baikal. At intermediate or decadal scales, the lake temperature time series reveals anomalies in the rotational velocity of the planet. And at the shortest time scales of 3 to 4 years, the time series reveals connections to pressure and temperature variations thousands of kilometers away—mediated by interactions with other climate patterns and apparently transduced by variability in the trajectory of the jet stream. All of these signals are present in the Lake Baikal data simultaneously in different bandwidths, and all may influence the lake's ecology.

## Materials and Methods

### Overarching approach

Seasonality of annual water temperature change was captured as phase (*Φ*) of the annual harmonic estimated *via* Short-Time Fourier Transform (STFT), calculated at multiple windows of time in the water temperature time series in order to examine how seasonality changed across finer and broader scales of variability related to climate. A time series of phase was generated (Fig. 1) using the moving window of STFT. This time series, or subsets of it, were then cross-correlated with similar portions of time series of climate indexes. In all cases, selection of these subsets was based on prospective relationships suggested by existing literature (e.g. PDO eras). In most cases, temporal autocorrelation in the raw data necessitated filtering (“pre-whitening”) before analysis, to avoid the emergence of spurious relationships that can occur between time series with strong autocorrelation [47]. Cross-correlations between these pre-whitened phase time series and climate indices allowed examination of both short- and long-term seasonality changes.

### Lake Baikal data

Data were collected on a schedule of 10–14 days at the same location on Lake Baikal, 2.7 km from the village of Bol'shie Koty at 51.9018°N and 105.0665°E (maximum depth 800 m) from January 1946 to December 2003 [14]. The time series are constructed of monthly mean values assuming that all months are the same length (Δt = 0.083^ ^^ year). In the entire 58 year data set, missing values amounted to 6.3% of the time series. For the time series analysis, missing values were replaced with the 58-year mean for that month.

### Generating the phase time series from water temperature data

The long-term, linear, increasing trend in surface temperature [16] was removed before time-frequency analysis. Short segments, or windows of the de-trended time series were transformed into the frequency domain via Fourier transform in segments that ranged from n = 36 to 276 months. Estimation of Short-Time Fourier Transform spectra was performed with LabVIEW ver. 8.5 2007 (National Instruments, Austin, TX.). Magnitude and phase (*Φ*) for the frequency (*ω*) expressing the annual seasonality was identified in the transform (*ω* = 0.083^ ^^ month^−1^). Time series of different segment lengths were mean-padded to generate spectral resolution necessary to estimate the annual seasonality. The estimation was repeated as the window was slid along the time series by one month, with each window overlapping the previous. The estimate of phase for each segment of length *n* was referenced to the mid point of that segment in time (*t*), and the estimates were assembled into a sequential time series of *Φ*(*t,n*). Thus each estimate of the phase of the annual harmonic in the time series was made independently, but autocorrelation in the data means that adjacent estimates carry common information. The estimation of the Fourier spectra was performed simply to make a quantitative estimate of the deviation of the characteristic annual fluctuation in lake temperature from a stationary signal. Clearly there is much additional information in the unreferenced harmonics in the estimated spectra, but evaluating this was not the objective of the current study.

### Climate indices

In this study we compared the *Φ*(*t,n*) time series to various climate indexes. We examined Length of Day (LOD), Arctic Oscillation (AO), El Niño Southern Oscillation (ENSO), and Pacific Decadal Oscillation (PDO) as indices that may relate large-scale climate processes to abiotic monitoring data at Lake Baikal. Climate index monthly time series were obtained from the following public data servers: AO - Joint Institute for the Study of Atmosphere and Ocean (http://jisao.washington.edu/ao/#data); Global-SST ENSO index - Joint Institute for the Study of Atmosphere and Ocean (http://jisao.washington.edu/data/globalsstenso/#digital_values); PDO - Joint Institute for the Study of Atmosphere and Ocean (http://jisao.washington.edu/data/pdo/#data); LOD - International Earth Rotation Reference System Services Frankfurt, Germany (http://www.iers.org/). AO, ENSO and PDO are reported as monthly indexes; LOD is reported as 6-hour estimates. LOD raw values were averaged for each month to produce a monthly average before further signal processing.

### Data transformations

All of the monthly indexes had significant, but widely-varying degrees of first-order autocorrelation. For example, the *Φ*(*t*,40) time series power spectrum had only 1.7% of the temporal variance contributed by harmonics higher than the annual harmonic, whereas the raw Global-SST ENSO index had 9% of the temporal variance contributed by the same harmonics. Relevant comparisons between time series required multiple approaches to filtering the data. On the one hand, to evaluate the possible presence of a statistically significant cross-correlation in the data, raw *Φ*(*t*,n) and raw climate indices were filtered with a process that introduced noise with a uniform (white) distribution, “pre-whitening” [47]. The amplitude of the noise was increased until the first order autocorrelation in the time series fell below significance for the given length of time. Since the raw Global-SST ENSO signal contained higher frequency components, the amplitude of noise added to the raw *Φ*(*t*,40) was greater than the Global-SST ENSO index. On the other hand, when comparing time series that maintained their autocorrelation structures we smoothed the raw, monthly climate index time series with a 5-point finite impulse response filter until both series being compared had similar spectral energy above the annual harmonic. Significant energy above the annual harmonic is perceived as noise or uncorrelated error in the original time series [47]. Cross correlation comparisons between time series with widely divergent high frequency character (i.e. different levels of independence of errors) can lead to spurious inferences simply because the errors may not be independent [48]. As a consequence, each climate time series existed in three forms: “raw” values, “pre-whitened”, and “smoothed” values in order to compare apples-to-apples in each case.

### Cross-correlations

Temporal cross-correlations between *Φ*(*t*,n) and various climate indices were then performed in STATGRAPHICS Plus for Windows 3.0. Bonferroni adjustment was used to maintain an experiment-wise α = 0.05 with multiple comparisons, such that α_a_ = 0.002.
